# Supplementary material for: PARP14 is a writer, reader, and eraser of mono-ADP-ribosylation
Source: J Biol Chem. 2023 Jul 26;299(9):105096. doi: 10.1016/j.jbc.2023.105096 (PMC10470015; doi:10.1016/j.jbc.2023.105096)
Supplement: Supporting information [file mmc1.pdf]

Supporting information accompanying:

PARP14 is a writer, reader and eraser of mono-ADP-ribosylation

Archimede Torretta, Constantinos Chatzicharalampous, Carmen Ebenwaldner, Herwig Schüler*

Center for Molecular Protein Science (CMPS), Department of Chemistry, Lund University, 22100 Lund, Sweden

*herwig.schuler@biochemistry.lu.se

This file includes:

Supplementary Experimental Procedures
Supplementary Figure 1
Supplementary Figure 2
Supplementary Figure 3
Supplementary Figure 4
Supplementary Figure 5
Supplementary Figure 6

Supplementary Experimental Procedures

Thermal stability and ligand induced T_m shift analysis by differential scanning fluorimetry

Melting point (T_m) assays were performed in white 96-well PCR plates (Bio-Rad #MLL9651). Every well contained a 25 µL solution of 0.4 mg/mL of enzyme, 2 mM of Z5010894420, Z5014193706, or Z5183357278 (Enamine), and SyproOrange (ThermoFisher; at 1:5000 dilution) in 50 mM HEPES pH 7.5, 300 mM NaCl, 10 %v/v glycerol, 0.5 mM TCEP buffer. Control wells contained either 0, 2, or 8% DMSO. Plates were incubated at 20 ºC for 10 seconds and then the temperature was increased by 1 ºC/min up to 95 ºC. Fluorescence signals were measured with the CFX96 Touch Real-Time PCR Detection System (Bio-Rad) and data analysis was performed with the CFX Manager software (version 3.1, Bio-Rad).

PARP14 macrodomain-1 compound inhibition assay

ADP-ribosyltaion reactions were performed as described in the main text. 50 µL per well of solutions of PARP14 macrodomain-1 at a final concentration of 3 µM and different concentrations (10, 50, 100, 500 and 1000 µM) of SARS-CoV-2 Nsp3 Mac1 inhibitors (Enamine) or 1, 2, or 4% DMSO were added, except for positive control (omission of macrodomain), negative control (omission of NAD^+) and blank, where 50 µL of 50 mM HEPES pH 7.5, 100 mM NaCl, 0.2 mM TCEP, 4 mM $MgCl_2$, 0.1 mM EDTA buffer was added. The plates processed and analyzed as stated in the main text.

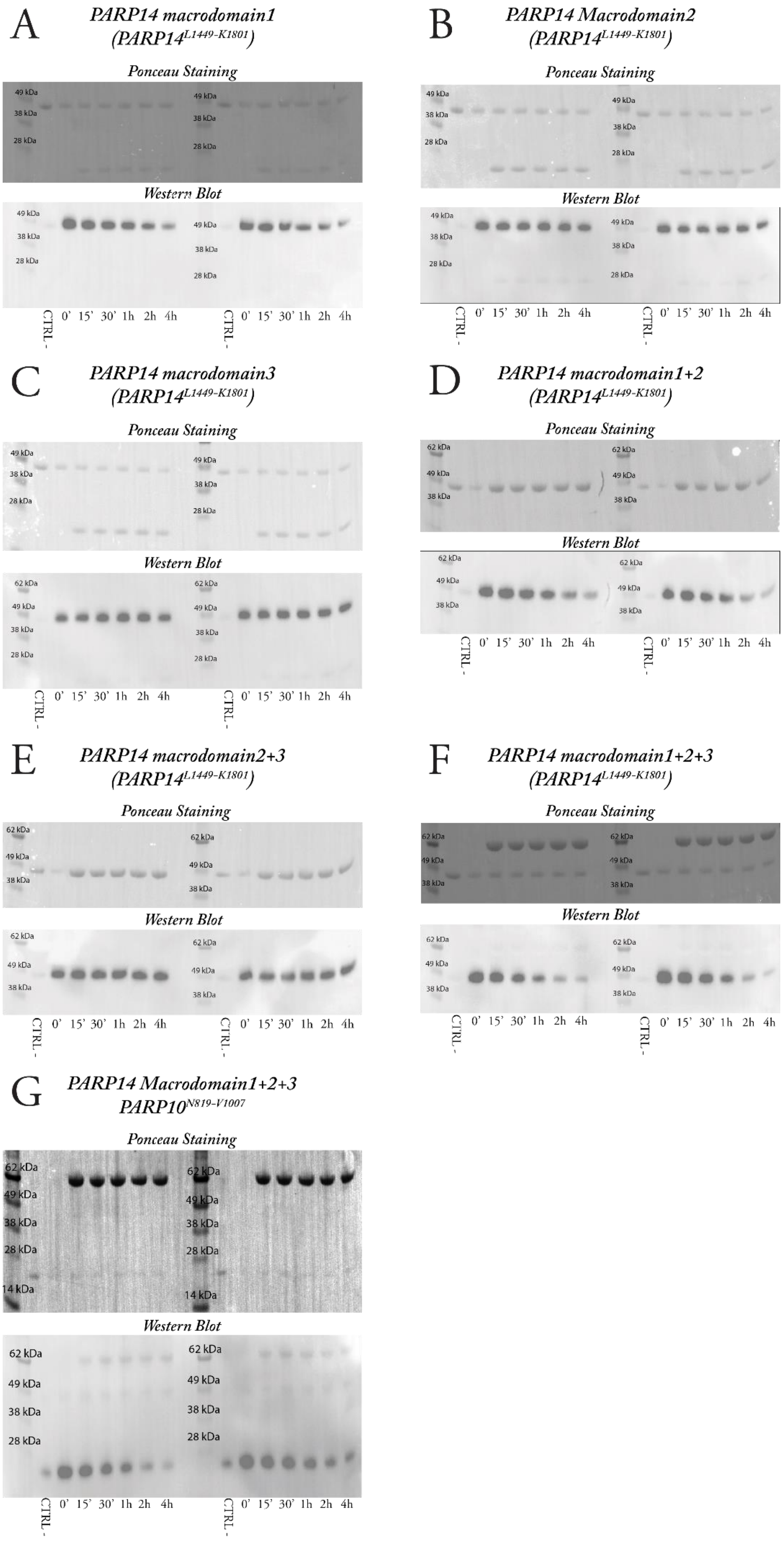
A
PARP14 macrodomain1
(PARP14L1449-K1801)
Ponceau Staining
Western Blot
49 kDa
38 kDa
28 kDa
CTRL -
0' 15' 30' 1h 2h 4h
B
PARP14 Macrodomain2
(PARP14L1449-K1801)
Ponceau Staining
Western Blot
C
PARP14 macrodomain3
(PARP14L1449-K1801)
Ponceau Staining
Western Blot
62 kDa
D
PARP14 macrodomain1+2
(PARP14L1449-K1801)
Ponceau Staining
Western Blot
E
PARP14 macrodomain2+3
(PARP14L1449-K1801)
Ponceau Staining
Western Blot
F
PARP14 macrodomain1+2+3
(PARP14L1449-K1801)
Ponceau Staining
Western Blot
G
PARP14 Macrodomain1+2+3
PARP10N819-V1007
Ponceau Staining
Western Blot
14 kDa

***Previous page* Figure S1** (related to Fig. 1 of the main text)

A-G Original membranes, including duplicate reactions and molecular weight markers, of the experiments shown in Fig. 1 of the main text. Experiments not shown in the main text are those pertaining PARP14 macrodomain-2 and -3 (panels B and C, respectively).

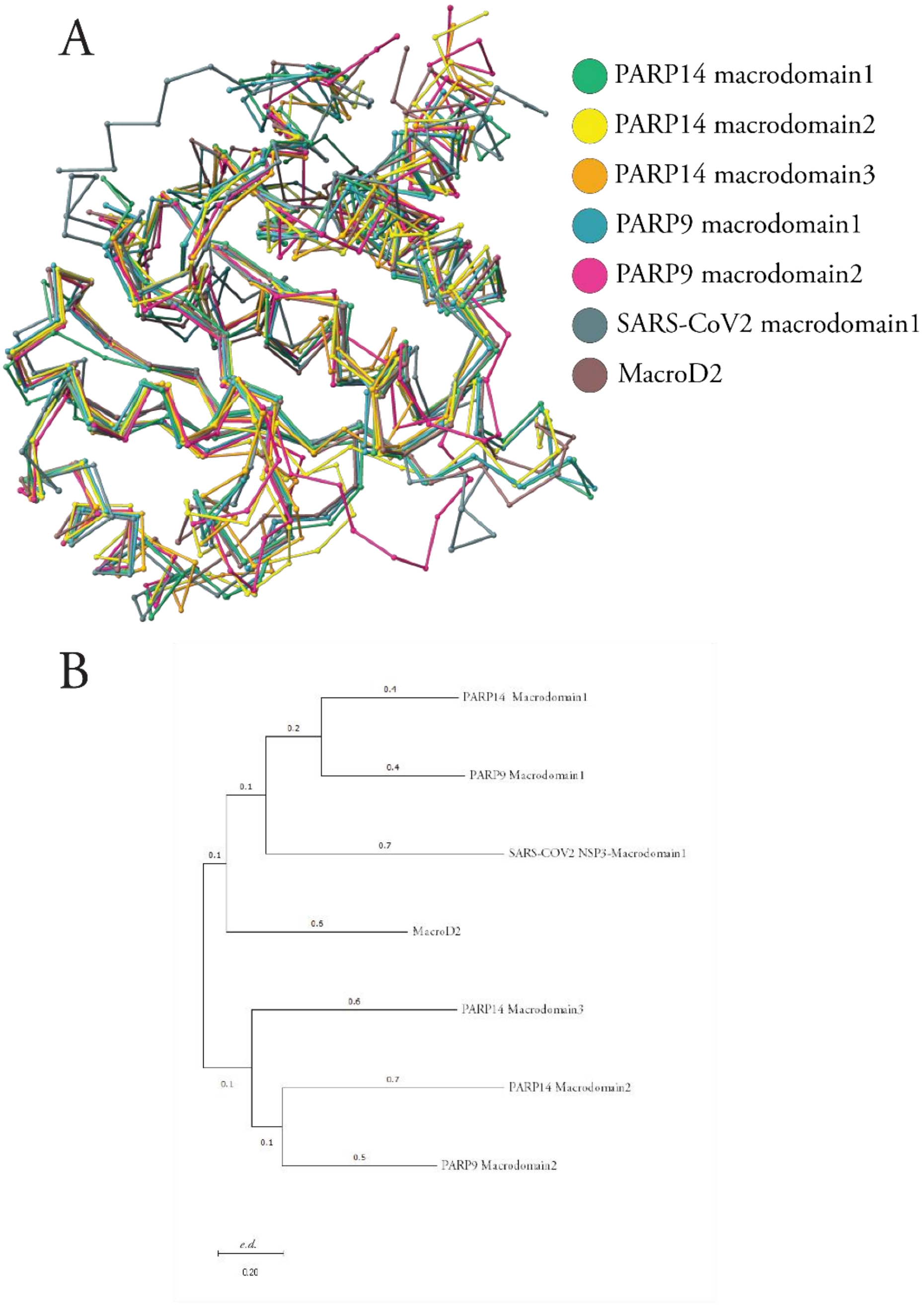

Figure S2 (related to Fig. 2 of the main text)

A Alignment of the crystal structures of the macrodomains shown in the sequence alignment of Fig.1B of the main text. **B** Phylogenetic tree of the same sequences calculated using MEGA11.

Table 1. Identity and similarity scores.

	PARP14 macrodomain1	PARP9 macrodomain1	PARP14 macrodomain2	PARP9 macrodomain2	PARP14 macrodomain3	macroD2	NSP3 macrodomain1	
**PARP14 macrodomain1**	-	54.25%	33.51%	29.67%	35.46%	37.79%	40.65%	Similarity
**PARP9 macrodomain1**	42.02%	-	34.04%	28.57%	33.72%	37.20%	40.65%	
**PARP14 macrodomain2**	22.87%	21.8%	-	43.95%	34.88%	23.25%	32.41%	
**PARP9 macrodomain2**	15.93%	17.03%	31.31%	-	40.69%	25.58%	32.41%	
**PARP14 macrodomain3**	21.51%	19.18%	22.67%	31.39%	-	25.00%	33.72%	
**macroD2**	23.83%	26.16%	15.69%	18.02%	14.53%	-	34.30%	
**NSP3 macrodomain1**	29.12%	26.37%	20.32%	23.62%	23.83%	22.09%	-	
				Identity				

Table 2. Cα RMSD values* calculated from the structural alignment in Figure 1 B.

	PARP14 macrodomain2	PARP14 macrodomain3	PARP9 macrodomain1	PARP9 macrodomain2	NSP3 macrodomain1	macroD2
**PARP14 macrodomain1**	1.460	1.333	0.546	1.705	1.331	0.721
**PARP14 macrodomain2**	-	1.243	1.220	1.055	4.328	1.167
**PARP14 macrodomain3**	-	-	1.187	1.097	6.415	1.194
**PARP9 macrodomain1**	-	-	-	1.146	1.968	0.798
**PARP9 macrodomain2**	-	-	-	-	1.346	1.321
**NSP3 macrodomain1**	-	-	-	-	-	1.832

***Alignment RMSD values are expressed in Å**

A

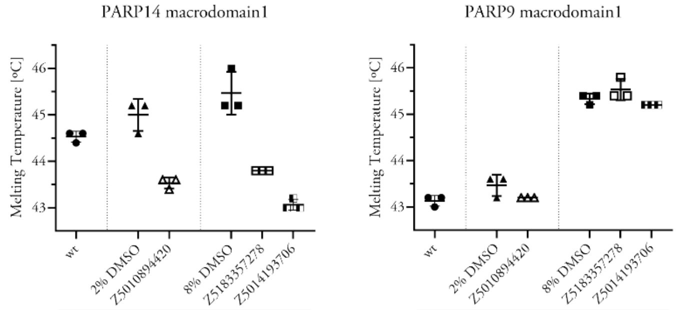

	Mean	Std. Deviation
wt	44.53	0.1155
2% DMSO	45.00	0.3464
Z5010894420	43.53	0.1155
8% DMSO	45.47	0.4619
Z5183357278	43.80	0.000
Z5014193706	43.07	0.1155

	Mean	Std. Deviation
wt	43.13	0.1155
2% DMSO	43.47	0.2309
Z5010894420	43.20	0.000
8% DMSO	45.33	0.1155
Z5183357278	45.53	0.2309
Z5014193706	45.20	0.000

B

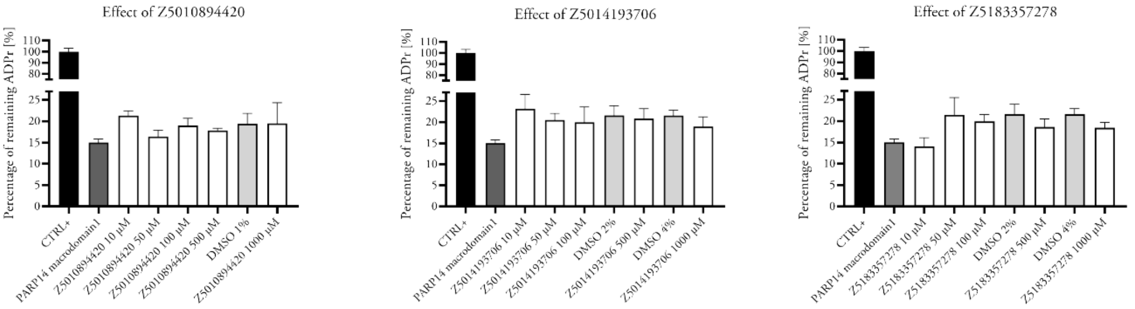

Figure S3

Effect of SARS-CoV-2 Nsp3 Mac1 inhibitors on PARP14 and PARP9 macrodomain-1. A Melting point shift assays of PARP14 (left) respectively PARP9 (right) macrodomain-1 in the presence of 2 mM of the indicated compounds. The effect of DMSO, at the concentrations required for this concentration for the respective compounds, is also shown. All three compounds destabilized PARP14 macrodomain-1 and had no effect on the T_m of PARP9 macrodomain-1. **B** Inhibition assays (using detection of remaining ADP-ribosylation levels using MacroGreen) of PARP14 macrodomain-1 activity over automodified PARP14. The relevant concentrations of DMSO are shown in light grey bars. None of the compounds significantly inhibited macrodomain-1.

Next page **Figure S4** (related to Fig. S3)

Effect of SARS CoV 2 Nsp3 Mac1 inhibitors on PARP14 and PARP9 macrodomain-1. Left panels, thermal melting profiles; right hand panels, first derivatives.

A

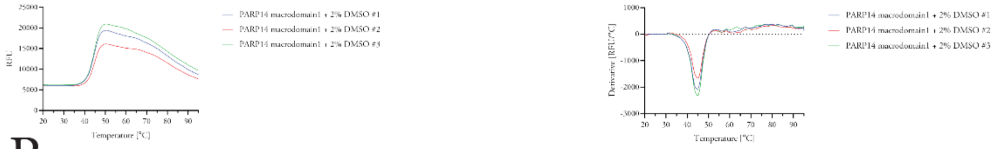
RFU
Temperature [°C]
PARP14 macrodomain1 + 2% DMSO #1
PARP14 macrodomain1 + 2% DMSO #2
PARP14 macrodomain1 + 2% DMSO #3
Derivative [RFU/°C]
Temperature [°C]
PARP14 macrodomain1 + 2% DMSO #1
PARP14 macrodomain1 + 2% DMSO #2
PARP14 macrodomain1 + 2% DMSO #3

B

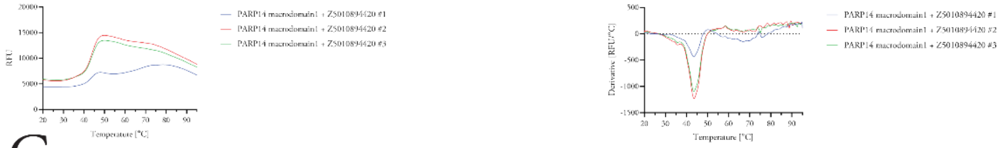
RFU
Temperature [°C]
PARP14 macrodomain1 + Z5010894420 #1
PARP14 macrodomain1 + Z5010894420 #2
PARP14 macrodomain1 + Z5010894420 #3
Derivative [RFU/°C]
Temperature [°C]
PARP14 macrodomain1 + Z5010894420 #1
PARP14 macrodomain1 + Z5010894420 #2
PARP14 macrodomain1 + Z5010894420 #3

C

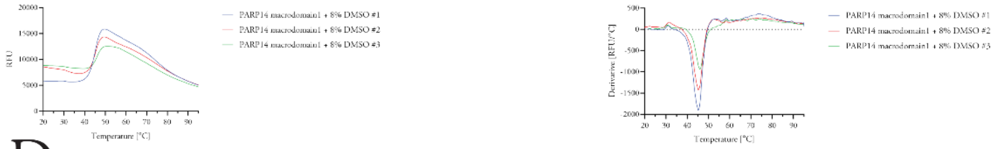
RFU
Temperature [°C]
PARP14 macrodomain1 + 8% DMSO #1
PARP14 macrodomain1 + 8% DMSO #2
PARP14 macrodomain1 + 8% DMSO #3
Derivative [RFU/°C]
Temperature [°C]
PARP14 macrodomain1 + 8% DMSO #1
PARP14 macrodomain1 + 8% DMSO #2
PARP14 macrodomain1 + 8% DMSO #3

D

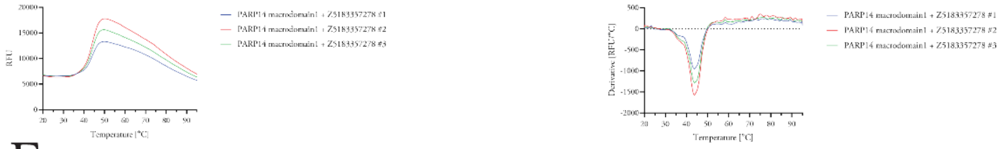
RFU
Temperature [°C]
PARP14 macrodomain1 + Z5183357278 #1
PARP14 macrodomain1 + Z5183357278 #2
PARP14 macrodomain1 + Z5183357278 #3
Derivative [RFU/°C]
Temperature [°C]
PARP14 macrodomain1 + Z5183357278 #1
PARP14 macrodomain1 + Z5183357278 #2
PARP14 macrodomain1 + Z5183357278 #3

E

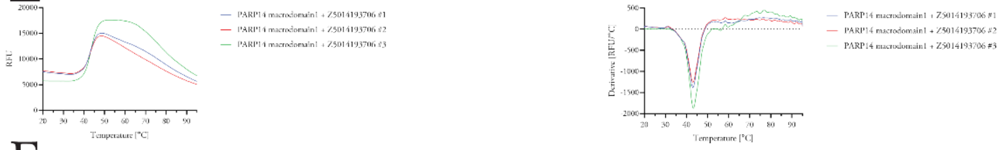
RFU
Temperature [°C]
PARP14 macrodomain1 + Z5014193706 #1
PARP14 macrodomain1 + Z5014193706 #2
PARP14 macrodomain1 + Z5014193706 #3
Derivative [RFU/°C]
Temperature [°C]
PARP14 macrodomain1 + Z5014193706 #1
PARP14 macrodomain1 + Z5014193706 #2
PARP14 macrodomain1 + Z5014193706 #3

F

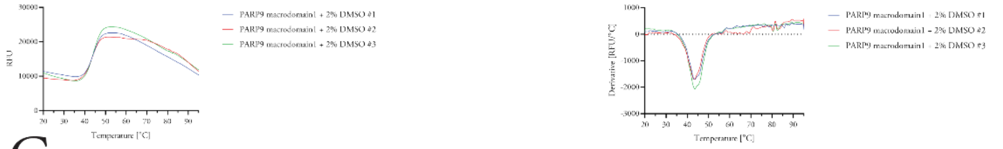
RFU
Temperature [°C]
PARP9 macrodomain1 + 2% DMSO #1
PARP9 macrodomain1 + 2% DMSO #2
PARP9 macrodomain1 + 2% DMSO #3
Derivative [RFU/°C]
Temperature [°C]
PARP9 macrodomain1 + 2% DMSO #1
PARP9 macrodomain1 + 2% DMSO #2
PARP9 macrodomain1 + 2% DMSO #3

G

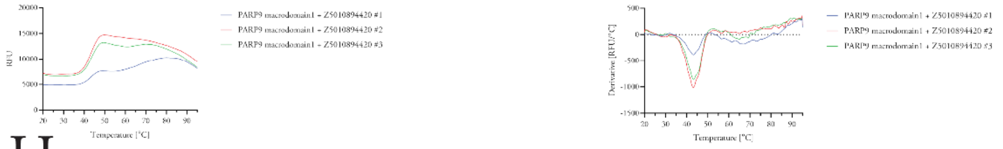
RFU
Temperature [°C]
PARP9 macrodomain1 + Z5010894420 #1
PARP9 macrodomain1 + Z5010894420 #2
PARP9 macrodomain1 + Z5010894420 #3
Derivative [RFU/°C]
Temperature [°C]
PARP9 macrodomain1 + Z5010894420 #1
PARP9 macrodomain1 + Z5010894420 #2
PARP9 macrodomain1 + Z5010894420 #3

H

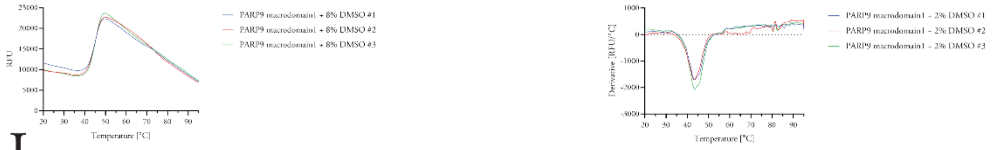
RFU
Temperature [°C]
PARP9 macrodomain1 + 8% DMSO #1
PARP9 macrodomain1 + 8% DMSO #2
PARP9 macrodomain1 + 8% DMSO #3
Derivative [RFU/°C]
Temperature [°C]
PARP9 macrodomain1 + 2% DMSO #1
PARP9 macrodomain1 + 2% DMSO #2
PARP9 macrodomain1 + 2% DMSO #3

I

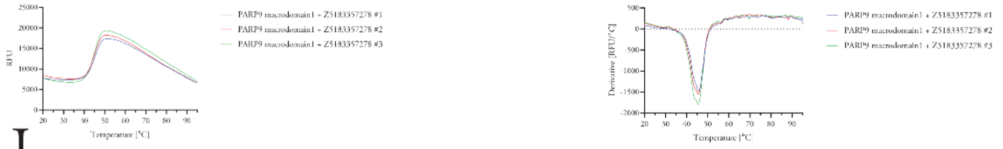
RFU
Temperature [°C]
PARP9 macrodomain1 + Z5183357278 #1
PARP9 macrodomain1 + Z5183357278 #2
PARP9 macrodomain1 + Z5183357278 #3
Derivative [RFU/°C]
Temperature [°C]
PARP9 macrodomain1 + Z5183357278 #1
PARP9 macrodomain1 + Z5183357278 #2
PARP9 macrodomain1 + Z5183357278 #3

J

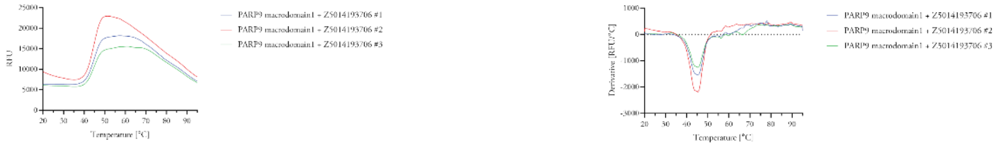
RFU
Temperature [°C]
PARP9 macrodomain1 + Z5014193706 #1
PARP9 macrodomain1 + Z5014193706 #2
PARP9 macrodomain1 + Z5014193706 #3
Derivative [RFU/°C]
Temperature [°C]
PARP9 macrodomain1 + Z5014193706 #1
PARP9 macrodomain1 + Z5014193706 #2
PARP9 macrodomain1 + Z5014193706 #3

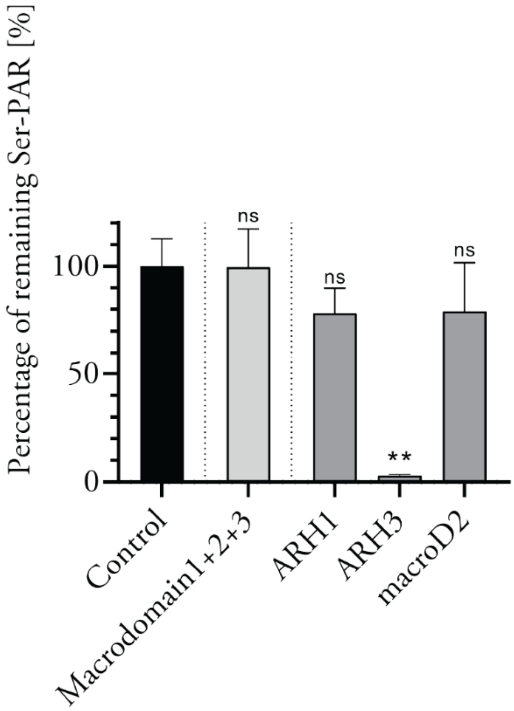

Figure S5 (related to Fig. 3 of the main text)

Quantification using MacroGreen of ADP-ribosylation levels present after incubation of PARP1 with HPF1 and NAD+ and subsequent incubation with the indicated macrodomains. Error bars represent S.D. (n=4) and significance levels refer to the control.

Next page **Figure S6** (related to Fig. 4 of the main text)

Differential Scanning Fluorimetry (DSF) of PARP14 and PARP9 macrodomain-1 wild type and F->A mutants in presence and absence of free ADP-ribose. Left panels, thermal melting profiles; right hand panels, first derivatives.

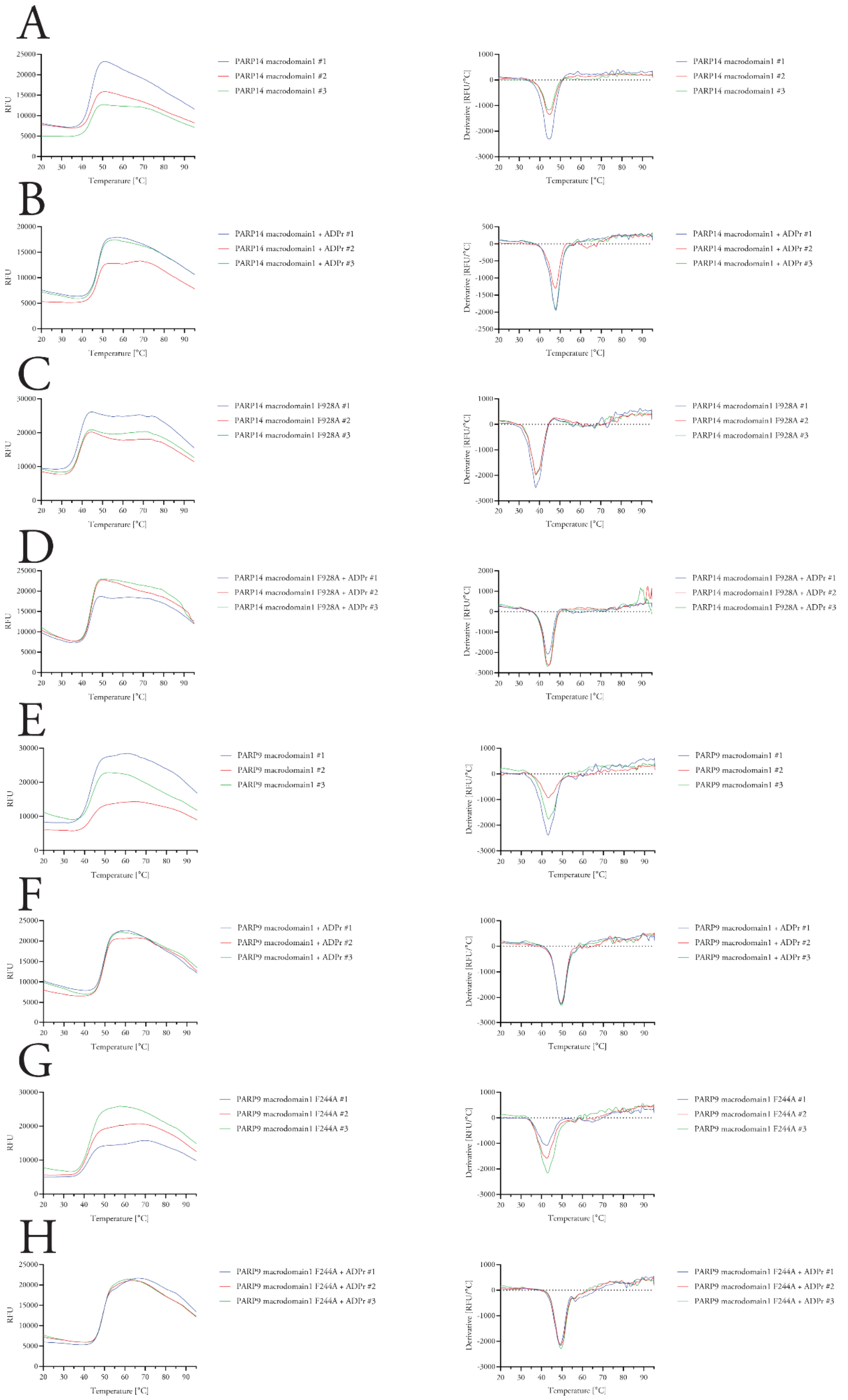
A
RFU
Temperature [°C]
PARP14 macrodomain1 #1
PARP14 macrodomain1 #2
PARP14 macrodomain1 #3
Derivative [RFU/°C]
Temperature [°C]
PARP14 macrodomain1 #1
PARP14 macrodomain1 #2
PARP14 macrodomain1 #3
B
RFU
Temperature [°C]
PARP14 macrodomain1 + ADPr #1
PARP14 macrodomain1 + ADPr #2
PARP14 macrodomain1 + ADPr #3
Derivative [RFU/°C]
Temperature [°C]
PARP14 macrodomain1 + ADPr #1
PARP14 macrodomain1 + ADPr #2
PARP14 macrodomain1 + ADPr #3
C
RFU
Temperature [°C]
PARP14 macrodomain1 F928A #1
PARP14 macrodomain1 F928A #2
PARP14 macrodomain1 F928A #3
Derivative [RFU/°C]
Temperature [°C]
PARP14 macrodomain1 F928A #1
PARP14 macrodomain1 F928A #2
PARP14 macrodomain1 F928A #3
D
RFU
Temperature [°C]
PARP14 macrodomain1 F928A + ADPr #1
PARP14 macrodomain1 F928A + ADPr #2
PARP14 macrodomain1 F928A + ADPr #3
Derivative [RFU/°C]
Temperature [°C]
PARP14 macrodomain1 F928A + ADPr #1
PARP14 macrodomain1 F928A + ADPr #2
PARP14 macrodomain1 F928A + ADPr #3
E
RFU
Temperature [°C]
PARP9 macrodomain1 #1
PARP9 macrodomain1 #2
PARP9 macrodomain1 #3
Derivative [RFU/°C]
Temperature [°C]
PARP9 macrodomain1 #1
PARP9 macrodomain1 #2
PARP9 macrodomain1 #3
F
RFU
Temperature [°C]
PARP9 macrodomain1 + ADPr #1
PARP9 macrodomain1 + ADPr #2
PARP9 macrodomain1 + ADPr #3
Derivative [RFU/°C]
Temperature [°C]
PARP9 macrodomain1 + ADPr #1
PARP9 macrodomain1 + ADPr #2
PARP9 macrodomain1 + ADPr #3
G
RFU
Temperature [°C]
PARP9 macrodomain1 F244A #1
PARP9 macrodomain1 F244A #2
PARP9 macrodomain1 F244A #3
Derivative [RFU/°C]
Temperature [°C]
PARP9 macrodomain1 F244A #1
PARP9 macrodomain1 F244A #2
PARP9 macrodomain1 F244A #3
H
RFU
Temperature [°C]
PARP9 macrodomain1 F244A + ADPr #1
PARP9 macrodomain1 F244A + ADPr #2
PARP9 macrodomain1 F244A + ADPr #3
Derivative [RFU/°C]
Temperature [°C]
PARP9 macrodomain1 F244A + ADPr #1
PARP9 macrodomain1 F244A + ADPr #2
PARP9 macrodomain1 F244A + ADPr #3
